# Impaired uterine artery flow associated with the presence of ovarian endometrioma: preliminary results of a prospective study

**DOI:** 10.1186/1757-2215-7-1

**Published:** 2014-01-08

**Authors:** Maria Grazia Porpora, Federica Tomao, Lucia Manganaro, Deliar Yazdanian, Eliana Fuggetta, Maria Grazia Piccioni, Pierluigi Benedetti Panici, Giuseppe Benagiano

**Affiliations:** 1 Department of Gynecology, Obstetrics and Urology, Sapienza, University of Rome, Policlinico Umberto I, Viale Regina Elena 324, Rome 00161, Italy; 2 Department of Radiological Sciences, Oncology and Pathology, Sapienza, University of Rome, Policlinico Umberto I, Viale Regina Elena 324, Rome 00161, Italy

**Keywords:** Color doppler, Endometriosis, Infertility, Ultrasounds, Uterine artery flow, Resistance Index (RI)

## Abstract

**Background:**

Aim of this prospective, case–control study was to evaluate uterine arteries’ blood flow before and after laparoscopic surgery in patients with ovarian endometriosis and its possible correlation with infertility.

**Methods:**

We prospectively enrolled 110 women of reproductive age; 69 with ovarian endometriomas and scheduled for surgery, and 41 controls. At enrolment, a detailed medical, gynecologic and obstetric history was collected. Fertility and pregnancy desire were assessed. All patients underwent complete physical and gynecologic examination. Transvaginal ultrasound with Doppler color flow was performed to evaluate Resistance Index (RI) of uterine arteries during the secretory phase, at enrolment (T0) and 3 months after laparoscopic surgery (T1).

**Results:**

Among cases, 27 patients were excluded because they did not meet the inclusion criteria. At enrolment (T0) unilateral or bilateral flow alterations (RI ≥ 0.8) were found in 38 out of 42 patients with ovarian endometriosis (90%), whereas in the control group only 17 women (41%) had Doppler alterations. The difference in uterine artery RI values between cases and controls was statistically significant (P < 0.0001). A statistically significant improvement in uterine artery flow (P <0.0001) was found 3 months after surgical treatment of endometriosis. Nineteen patients with endometriosis (45%) were infertile before surgery; all of them presented uterine artery Doppler alterations at T0. After surgery the pregnancy rate was significantly higher in patients who presented uterine artery flow normalization than in those with persistent uterine artery flow alterations (p = 0.002).

**Conclusions:**

A strong correlation was found between uterine artery flow abnormalities and ovarian endometriosis. Uterine artery flow improvement following surgery seems to increase the probabilities of achieving pregnancy.

## Background

The presence of endometriosis induces a chronic inflammatory condition capable of influencing pregnancy outcome [1] and, although the condition can be asymptomatic, according to a recent statement by the American Society for Reproductive Medicine "*typically presents with pelvic pain, infertility, or an adnexal mass, and may require surgery*" [2]. The prevalence of endometriosis in women undergoing laparoscopy for evaluation of infertility has been estimated between 9 and 50% and its presence is 6 to 8 times more frequent in infertile women [3].

A causal relationship between endometriosis and infertility has not been definitely established. In fact while in advanced stages the presence of adhesions and distorted pelvic anatomy may be sufficient to impair fertility, in earlier stages the relationship between endometriosis and subfertility is still a controversial issue. Modifications in the peritoneal fluid and pelvic inflammation may affect fertility and both oocyte and embryo quality. Moreover, eutopic endometrium is altered in women with endometriosis and possibly less receptive due to a well-documented local production of estradiol, coupled with a certain degree of progesterone resistance [4-6].

According to prospective cohort studies in infertile women with moderate and severe endometriosis, following laparoscopic removal of lesions and adhesiolysis, the crude spontaneous pregnancy rate ranges between 52 and 69% [7-9].

In the presence of an ovarian endometrioma additional specific factors may play a role: first of all, there is evidence that cystectomy may decrease the size of the follicle pool [10]; in addition, in these patients the age-dependent physiologic decline in the number of ovarian follicles can begin earlier in life [4] and, indeed, the presence of an endometrioma reduces ovarian reserve and decreases the number of oocytes retrieved [11,12]. *In vitro* fertilization (IVF) followed by embryo transfer (ET) has been extensively utilized to restore fertility in patients with endometriosis [13]. Unfortunately, there is an adverse effect of endometriosis on ovulation rates, ovarian reserve, and response to ovarian stimulation [14]. This effect has been attributed to the disease itself [11], although opinions on this point differ [15-17].

The work of Steer et al. [15] added a new dimension to the search for pathogenetic mechanisms of infertility, when they found cyclic variations in the uterine and ovarian artery blood flow throughout the menstrual cycle [15]. Steer et al. [16] then proposed that deviation from these physiologic changes might reduce fertility, since increased flow resistance in the uterine artery during the midluteal phase was found to be associated with unexplained infertility [16]. Recently El-Mazny et al. confirmed that uterine artery flow parameters were significantly altered in infertile patients, concluding that Doppler study of uterine hemodynamics should be considered in infertility work-up [18].

There are no studies in the literature evaluating uterine artery blood flow alterations in patients with endometriosis.

To explore this new field, a study was set-up to evaluate the presence of uterine artery blood flow alterations in women with ovarian endometriomas, its hypothetical normalization after surgery and a possible influence on reproductive outcome.

## Methods

One-hundred-ten women of reproductive age were enrolled in this prospective, case–control study: 69 consecutive patients with ovarian endometriomas who were scheduled for laparoscopic surgery and 41 controls selected among women undergoing routine check-up.

Patients were recruited among women referred to the Endometriosis and Chronic Pelvic Pain Center of the Department of Gynecology, Obstetrics and Urology of Sapienza, University of Rome, with a suspected diagnosis of ovarian endometrioma. From January 2012 to January 2013, a total of 69 patients with an ultrasound-determined diagnosis were recruited. Women who did not present with clinical and ultrasonographic signs of endometriosis, adenomyosis, history of infertility, uterine malformations and/or other ovarian pathologies and not taking any hormonal therapy, acted as controls; they were selected according to age-matched criteria.

The study protocol was approved by the Institutional Review Board of the University Hospital Azienda Policlinico Umberto I. Written informed consent was obtained from all patients and controls. Patients with endometriomas were enrolled in the study according to the following inclusion criteria:

1) Presence of an ultrasound (US) diagnosis of ovarian endometrioma(s) ≥ 4 cm.

2) Scheduled laparoscopy.

3) Age 18–45 years.

The exclusion criteria were: current hormonal treatment, presence of uterine fibroids, imaging signs of adenomyosis, gynecologic malformations, autoimmune disorders, chronic diseases, coagulation disorders, previous surgery for endometriosis, history of gynecologic surgery or pelvic inflammatory disease. Twenty-seven of the 69 patients were excluded because they did not meet these criteria.

The US diagnosis of ovarian endometrioma was made in the presence of the following preset criteria: a unilocular mass with ground glass echogenicity and a color score between 1 and 3; or a unilocular mass with ground glass echogenicity and a papillary projection, a color score of 1 or 2 and no flow inside the papillary projection [19].

Color Doppler flow imaging evaluation of vessel distribution was performed in all cases to reduce the number of false positive findings in the diagnosis of endometrioma.

At enrollment a detailed medical, gynecologic and obstetric history was collected. Dysmenorrhea, dyspareunia and chronic pelvic pain were evaluated by a visual analogue scale (VAS) [20]. Intensity of symptoms was classified as none (0), mild (1-4), moderate (5-7), or severe (8-10). Fertility and pregnancy desire were assessed.

All patients underwent complete physical and gynecologic examination.

Ultrasound (US) with Color Doppler imaging evaluation was performed using a GE Medical Systems Voluson© 730 Expert Doppler ultrasound instrument (GE Healthcare, Waukesha, WI), equipped with 3D transabdominal (3.5 MHz) and transvaginal (5 MHz) probe for imaging and a pulsed Doppler system for blood flow analysis. Uterine arterial blood flow velocity waveforms were obtained by means of color flow imaging to identify ascending branch of the uterine artery on the left and right sides of the uterine isthmus. The sample volume was placed on the artery with an angle of about 0°. At least three consecutive, correctly imaged blood flow velocity waveforms were analyzed and the resistance index (RI = maximal systolic velocity – least diastolic velocity / maximal systolic velocity) was calculated.

Resistance Index (RI) of uterine artery was evaluated during the mid-luteal phase (20^th^ -22^nd^ day of the cycle, confirmed by endometrial thickness between 8 and 16 mm), by Transvaginal Doppler Ultrasound with color flow.

In the presence of clinical and US suspicion of adenomyosis or deep endometriosis, patients underwent 3D ultrasound (3D-US) and Magnetic Resonance Imaging (MRI). US and MRI signs of deep endometriosis consisted of the specific finding of posterior cul-de-sac (PCS) obliteration, as previously described [21,22]; uterosacral ligament (USL) involvement was suspected when increased and inhomogeneous thickness was associated with abnormal arciform appearance [23].

As shown by Kurjac et al. (1991) [24], under physiologic conditions uterine flow velocity has a RI of 0.88 +/-0.04 in the proliferative phase and starts to decrease the day before ovulation; a nadir of 0.84 +/-0.04 is reached on day 18 and the index remains at that level for the rest of the cycle. On the basis of Kurjac’s findings, values of RI < 0.80 were considered normal [24].

All patients with endometriomas ≥ 4 cm were scheduled for laparoscopic treatment according to current guidelines [25]. Laparoscopy was performed under general anesthesia.

At laparoscopy, the diagnosis of ovarian endometriosis was confirmed and the disease staged according to the revised classification of the American Society of Reproductive Medicine (rASRM) [2]. Excision by stripping of monolateral or bilateral endometriomas, adhesiolysis and excision, or coagulation of peritoneal lesions using bipolar forceps, were performed by the same surgeon (MGP) with more than 20 years of experience, according to a previously described technique [26].

Tubal patency was assessed by chromopertubation (Lap-Dye) in all patients. Histologic examination was also carried out in all cases.

Ultrasound examination with Doppler evaluation was performed at enrolment (T0) and 3 months after laparoscopy (T1) by two trained blinded physicians with 20 years of experience in pelvic ultrasound examinations. The median follow up was 7 (2-12) months.

All data obtained from both groups of patients were prospectively stored in an appropriate database.

Data were analyzed using the Statistical Package for Social Sciences (SPSS) version 18.

Parametric tests were carried out after having examined the normal distribution of data to be analyzed. The Student t test was used for continuous parametric variables. The Fisher exact test and the chi-squared test were used for categorical variables. A P value below 0.05 was considered statistically significant.

## Results

Among the 69 consecutive patients in whom a diagnosis of endometrioma was posed, only 42 met the inclusion criteria. In fact, 27 patients were excluded from the study: 16 were taking oral contraceptives, 3 presented also with uterine fibroids, 1 had uterine malformation, 2 had concomitant uterine adenomyosis and 5 had deep endometriosis nodules of the recto-vaginal septum confirmed by 3D-US and MRI.

Median age was 32 (range 20–45 years) in the group of patients with endometrioma and 30 (range 18–45 years) in the control group (P = NS). No correlation was found between age and uterine artery RI values.

Characteristics of patients with endometriomas are reported in Table 1.

**Table 1:** Characteristic of patients with endometriosis at T0

**Characteristics**	**Results**
	***Median (range)***
Age	32 (20–45) years
Endometriosis Symptoms	*Patients (%)*
No dyspareunia	28 (67%)
Dyspareunia	
Grade I (1–4 VAS)	4 (9%)
Grade II (5–7 VAS)	5 (12%)
Grade III (8–10 VAS)	5 (12%)
No dysmenorrhea	13 (31%)
Dysmenorrhea	
Grade I (1–4 VAS)	2 (5%)
Grade II (5–7 VAS)	10 (24%)
Grade III (8–10 VAS)	17 (40%)
No Chronic pelvic pain (CPP)	35 (83%)
CPP	
Grade I (1–4 VAS)	0
Grade II (5–7 VAS)	6 (14%)
Grade III (8–10 VAS)	1 (2%)
Fertility	*Patients (%)*
Fertile	11 (26%)
Primary infertility	17 (40%)
Secondary infertility	2 (5%)
Unknown (with no desire of pregnancy or not yet considered infertile)	12 (29%)

**Figure 1:**
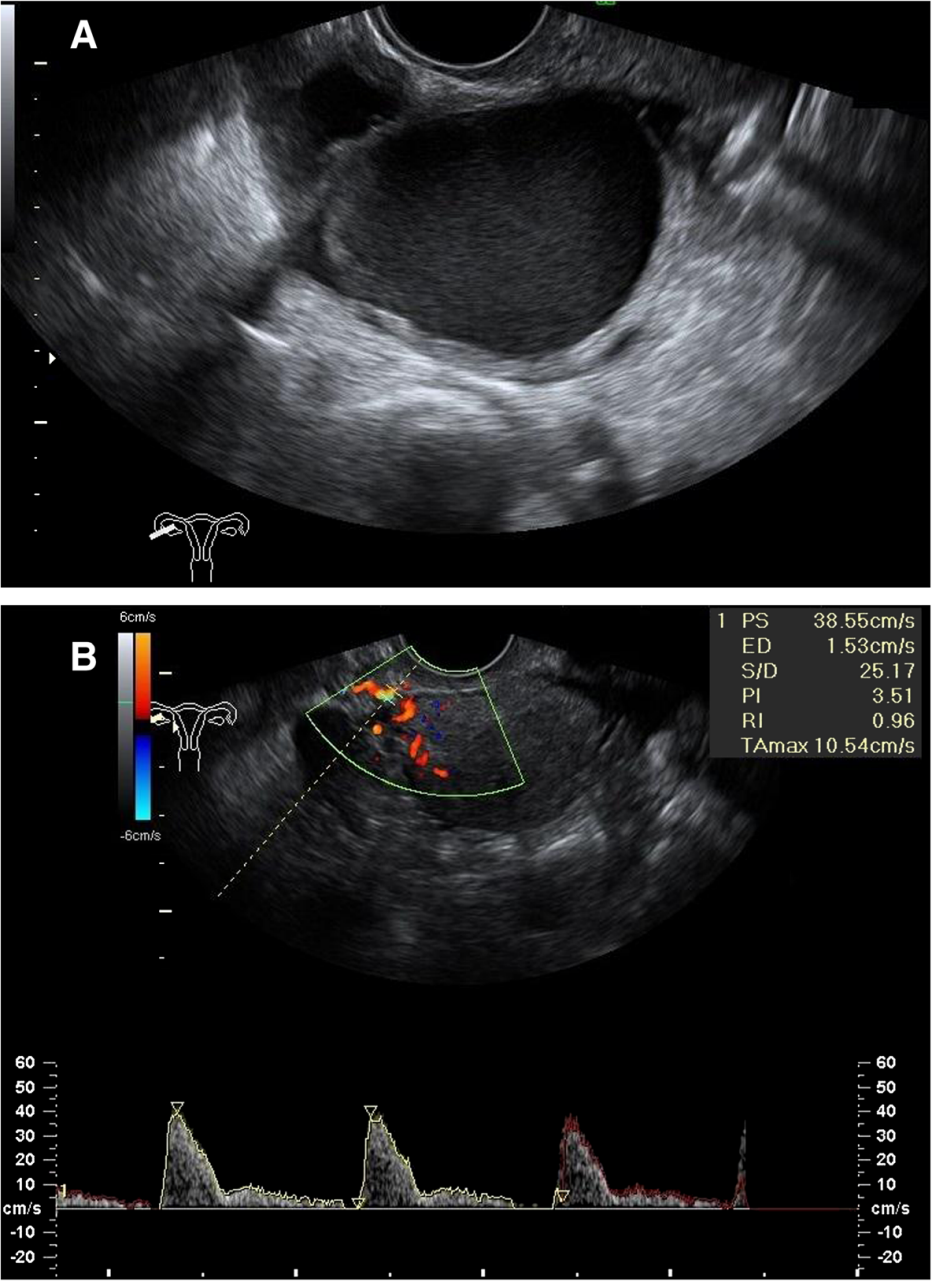
**Transvaginal ultrasonography and color Doppler. (A)** Right ovarian endometrioma and **(B)** Homolateral pre-operative Doppler flow alteration (RI = 0.96).

In 38 cases (90%), unilateral or bilateral flow alterations (RI ≥ 0.8) were observed, whereas only 4 patients showed a normal bilateral RI value. Among the 38 patients with Doppler alterations, 25 (66%) had bilateral and 13 (34%) unilateral increased RI values. The unilateral RI alteration coincided with the presence of homolateral endometrioma (Figure 1). All cases with infertility (primary in 17 cases and secondary in 2 cases) presented uterine artery flow alteration at T0.

In the control group, 17 subjects (41%) had Doppler alterations, whereas 24 (58%) had no abnormalities. Among the 17 women with Doppler alterations, 14 (82%) presented unilateral and only 3 (18%) bilateral high RI values.

A statistically significant difference between the median RI value in patients with endometrioma (0.84; range 0.6-1) and in the control group (0.76; range 0.48-0.97) was found (p <0.001).

Bilateral uterine artery blood flow alterations were significantly more frequent in the group of patients with endometrioma than in controls (p = 0.001).

Case–control comparison data are reported in Table 2.

**Table 2:** Ultrasonographic Doppler RI values (case–control comparison)

**Characteristics**	**Results**		
	***Median (range)***		**P value**
	**Case**	**Control**	
Median RI value	0.84 (0.6–1)	0.76 (0.48–0.97)	<0.001
	***Patients (%)***		
	**Case**	**Control**	
RI ≥0.8	38 (90%)	17 (41%)	<0.001
RI <0.8	4 (10%)	24 (59%)	
Unilateral RI alteration	13 (34%)	14 (82%)	0.001
Bilateral RI alteration	25 (66%)	3 (18%)	

At surgery, the presence of ovarian endometriosis was confirmed in all women; median size of the cyst was 43 mm (range 19–97 mm) in the right ovary and 38 mm (range 20–110 mm) in the left ovary, the overall median size was 40 mm (range 19–110 mm). All patients had at least 1 ovarian endometrioma ≥ 40 mm.

Bilateral ovarian endometriomas were found in 21 cases (50%), unilateral cysts in the remaining 21; 8 (38%) in the right ovary and 13 (62%) in the left ovary.

Adnexal adhesions were found in 28 cases (67%): adhesiolysis was performed in all patients. According to the rASRM classification, 18 women (43%) had stage III endometriosis and 24 (57%) had stage IV. No cases were classified as early stages of disease (I or II). No statistically significant correlation was found between rASRM stages III and IV and RI flow alterations (p = NS).

At chromopertubation (Lap-Dye) 15 (36%) patients had bilateral tubal patency, whereas 12 (28%) presented unilateral distal tubal occlusion (DTO), 10 (24%) unilateral proximal tubal occlusion (PTO) and 5 (12%) bilateral tubal occlusion (PTO in 1 patient and DTO in 4 women). Salpingoplasty was performed achieving tubal patency in 7 patients. No correlation was found between tubal status and uterine artery RI.

In all cases, histopathologic examination confirmed the diagnosis of endometrioma.

Among women with bilateral endometriomas, 14 (67%) had bilateral high RI values, whereas 5 (24%) had unilateral flow alterations and only 2 (9%) had no abnormalities.

Among patients with unilateral endometrioma 18 (85%) had homolateral flow alteration, only 1 (5%) had contralateral RI alteration and 2 (10%) had normal uterine artery flow.

Surgical findings in patients with endometrioma and their possible correlation with Doppler flow alterations are reported in Table 3.

**Table 3:** Surgical findings in patients with endometriosis and Doppler flow alterations (RI)

**Surgical findings**	**Results**			**P value**
***Cyst***				
	*Median (range)*			
**Median size**				NS
Overall	40 mm (10–110 mm)			
Right ovary	43 mm (19–97 mm)			
Left ovary	36 mm (10–110 mm)			
	*Patients (%)*			
**Maximum diameter**	Tot	RI ≥0.8	RI <0.8	
≥ 40 mm	32	28 (88%)	4 (12%)	NS
<40 mm	10	10 (100%)	/	
	*Patients (%)*			
**Site**	Tot	RI ≥0.8	RI <0.8	
Monolateral	21 (50%)	19 (90%)	2 (10%)	
(8 of the right ovary 13 of the left ovary)				NS
Bilateral	21 (50%)	19 (90%)	2 (10%)	
***r ASRM Stage***				
	*Patients (%)*			
	Tot	RI ≥0.8	RI <0.8	
III	18	17 (94%)	1 (6%)	NS
IV	24	21 (87%)	3 (13%)	
***Chromopertubation***				
	*Patients (%)*			NS
	RI ≥0.8	RI ≥0.8	RI <0.8	
Bilateral tubal patency	15 (36%)	12 (80%)	3 (20%)	
Tubal occlusion	27 (64%)	26 (96%)	1 (4%)	
Unilateral occlusion	22 (81%)			
Bilateral occlusion	5 (19%)			

No surgical findings were significantly correlated to Doppler flow values. Moreover, no statistically significant correlation was found between RI values and pain symptom intensity (dysmenorrhea, dyspareunia and chronic pelvic pain) evaluated by VAS score system (p = NS).

**Figure 2:**
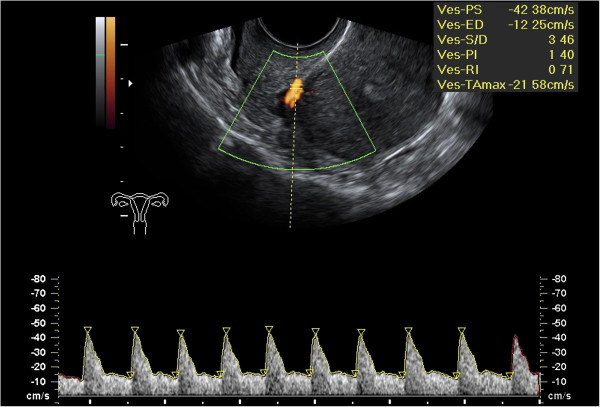
**Normalization of right uterine artery resistance index after laparoscopy (RI = 0.71) (the figure referred to the same patient reported in Figure**1**).**

Three months after surgery (T1), 23 (55%) patients had normal RI values (Figure 2), whereas 6 (14%) had bilateral and 13 (31%) unilateral flow alterations. In 19 out of 42 patients (45%) uterine RI artery flow persisted altered, even if in 7 cases a RI improvement was observed, although with values above normal.

There was a statistically significant difference between the number of patients with RI alterations at T0 (90%) and the number of patients with RI alterations at T1 (45%) (p < 0.001): these results are reported in Table 4.

**Table 4:** Post-operative Doppler modifications of uterine artery resistance index RI

	***Patients (%)***		**P value**
	**RI ≥ 0.8**	**RI < 0.8**	
Pre-operative	38 (90%)	4 (10%)	< 0.001
Post-operative	19 (45%)	23 (55%)	

Even if it was not the main objective of our study, we evaluated the reproductive outcome in terms of number of pregnancies during the limited follow-up period of a median of 7 months. Twenty-four patients (57%) desired pregnancy before surgery. Nineteen of them (79. 2%) were infertile (17 with primary and 2 with secondary infertility), and they all showed uterine artery Doppler alterations before surgery. Three months after laparoscopy, 14 (73.7%) out of 19 infertile patients had normal uterine artery RI flow, whereas RI alterations persisted in 5 cases.

Twelve out of the 19 (63%) infertile patients achieved a spontaneous pregnancy during the follow-up and all of them had normal RI values after surgery. Eight pregnancies (67%) occurred within the first 6 months and 4 (33%) within one year from laparoscopy. Five (41%) spontaneous pregnancies occurred in women showing a slow tubal passage of dye during laparoscopy; in 2 cases with a distal tubal obstruction a salpingoplasty was performed restoring tubal patency. The patient with bilateral proximal tubal occlusion underwent IVF-ET assisted reproductive technique with unfavorable fertility outcome.

A statistically significant difference in term of pregnancy rate was found between patients with normal RI values and those with persistent uterine artery flow alterations after surgery (p = 0.002). Pregnancy outcome is reported in Table 5.

**Table 5:** Pregnancy rate after surgery

	**Patients (%)**		**P-value**
Overall pregnancy rate	12 /42 (37%)		
Pregnancy rate in infertile patients	12/19 (63%)		
	**Pregnancies**	**No Pregnancies**	
Pregnancy rate according to post-operative Doppler values in infertile patients			
RI ≥ 0.8	0	5 (100%)	0.002
RI < 0.8	12 (86%)	2 (14%)	

Among the remaining 18 patients with no desire for pregnancy, 10 received hormonal contraception after surgery.

## Discussion

In the present study we observed that uterine artery RI was significantly altered in patients with ovarian endometriomas (RI value ≥0.8), showing higher uterine artery flow resistance compared with unaffected controls. In particular, all infertile patients presented uterine artery flow alteration at T0, confirming the possible role of uterine artery abnormalities in endometriosis-associated infertility.

Moreover, we observed that after laparoscopic treatment of the ovarian endometriomas, RI values returned to normal in the majority of patients. Uterine artery flow improvement was associated to a better fertility outcome with a percentage of spontaneous pregnancies of 67%; this rate is similar to post-operative pregnancy rate reported in the literature [27,28].

At present, surgical management of endometriomas in infertile women is the subject of debate, although there seems to be a growing consensus that these cysts should not be systematically removed.

Disagreement on the best treatment is predicated upon a series of conflicting findings. On the one hand, there is consistent evidence indicating that ovarian reserve is affected following surgical excision of ovarian endometriomas [29-32]. On the other, in infertile patients subjected to IVF without cyst excision, there is a risk of developing ovarian abscess following egg retrieval, due to contamination of the cyst content, as well as of rupture of the endometrioma. In addition, there may be difficulties in follicle aspiration, the risk of misdiagnosing an ovarian cancer and a growth of the cyst during pregnancy with a detrimental impact on its outcome [29,33-35].

Nevertheless data suggest that laparoscopic surgery is effective in the treatment of infertility associated with moderate to severe endometriosis, as long as resection is carefully carried out, avoiding substantial reduction of the ovarian reserve. Under these circumstances, excision of the cyst is associated with a subsequent increase in spontaneous pregnancy rate if compared with ablation [28,36-38].

Patients with endometriosis have been identified as having a variety of endocrine alterations that may impair the reproductive process and an altered uterine artery flow may be one. In these patients, luteal phase defects may be linked to decreased vascularization of the corpus luteum, due to the presence of the endometriotic cyst, with a consequent inadequate progesterone production; as such, it can be detected by studying ovarian and uterine artery flow dynamics. As previously mentioned, a recent paper published by El-Mazny et al. [18] highlighted that endometrial perfusion may play a role in the pathogenesis of infertility. The authors observed significantly more altered uterine artery flows in patients with unexplained infertility than in fertile parous controls. Moreover, since there were no significant differences in endometrial thickness and volume between the two groups, they hypothesized that the reduction in endometrial perfusion in the infertile group is more likely to reflect an aberrant end-organ effect of ovarian hormones on endometrial blood vessels. This may be due to suboptimal endometrial angiogenesis or dynamic vascular changes such as vasoconstriction or reduced vasodilatation [39].

Endometriosis is also associated with functional and structural changes in the eutopic endometrium and inner myometrium. The fact that we observed a strong correlation between uterine artery flow alteration and the presence of ovarian endometriosis supports the hypothesis that a defective blood supply may intervene in the overall mechanism of endometriosis-induced infertility, especially because this situation seems modifiable through surgical treatment. It is possible that the distortion of pelvic anatomy caused by the presence of endometriomas and/or the related inflammation could be responsible for uterine artery flow abnormalities which can be restored by surgical treatment. It is also possible that in the presence of an endometrioma associated to alterations in uterine artery blood flow, there are arteriosclerotic lesions in the endometrioma bed as suggested by Maneschi et al. [40] and/or endothelial dysfunction as suggested by Santoro et al. [40,41]. The present study has some limitations, first and foremost the relatively small number of cases and the short follow-up, even if most of the pregnancies after surgery for endometrioma usually occur within the first 6 months. Evaluation of reproductive outcome is still ongoing since a number of patients have not completed 1 year follow-up.

## Conclusions

Our results suggest that a careful evaluation of uterine artery flow may assume a role as prognostic factor of reproductive outcome after surgical treatment of ovarian endometrioma. Further studies are ongoing to confirm these preliminary data.

## Abbreviations

RI: Resistance index; IVF: in vitro fertilization; ET: Embryo transfer; US: Ultrasound; VAS: Visual analogue scale; MRI: Magnetic Resonance Imaging; PCS: Posterior Cul-de-sac; USL: Uterosacral ligament; rASRM: Revised classification of American Society of Reproductive Medicine; NS: Not significant; DTO: Distal tubal occlusion; PTO: Proximal tubal occlusion.

## Competing interests

All authors declare that they have no competing interest.

## Authors’ contributions

MGP, MGPi and PBP conceived the study and participated in its design. MGPi and LM performed the ultrasound examinations. MGP, FT, DY, EF and GB participated in the analysis and interpretation of data. DY, FT and EF collected the data. MGP, MGPi, GB, DY, FT, LM, PBP and EF wrote the paper. All authors read and approved the final manuscript.

## References

[B1] Petraglia F Arcuri F de Ziegler D Chapron C Inflammation: a link between endometriosis and preterm birth Fertil Steril 2012 98 1 36 40 10.1016/j.fertnstert.2012.04.051 22658345

[B2] Practice Committee of the American Society for Reproductive Medicine Endometriosis and infertility: a committee opinion Fertil Steril 2012 98 3 591 598 22704630 10.1016/j.fertnstert.2012.05.031

[B3] Rogers PA D’Hooghe TM Fazleabas A Giudice LC Montgomery GW Petraglia F Taylor RN Defining future directions for endometriosis research: workshop report from the 2011 World Congress of Endometriosis In Montpellier, France Reprod Sci 2013 20 5 483 499 10.1177/1933719113477495 23427182 PMC3635070

[B4] de Ziegler D Borghese B Chapron C Endometriosis and infertility: pathophysiology and management Lancet 2010 376 9742 730 738 10.1016/S0140-6736(10)60490-4 20801404

[B5] Macer ML Taylor HS Endometriosis and infertility: a review of the pathogenesis and treatment of endometriosis-associated infertility Obstet Gynecol Clin N Am 2012 39 4 535 549 10.1016/j.ogc.2012.10.002 PMC3538128 23182559

[B6] Carvalho LFP Rossener R Azeem A Malvezzi H Simões Abrão M Agarwal A From conception to birth: how endometriosis affects the development of each stage of reproductive life Minerva Ginecol 2013 65 2 181 198 23598783

[B7] Brosens I Gordts S Benagiano G Endometriosis in adolescents is a hidden, progressive and severe disease that deserves attention, not just compassion Hum Reprod 2013 28 8 2026 2031 10.1093/humrep/det243 23739215 PMC3712662

[B8] Loh FH Tan AT Kumar J Ng SC Ovarian response after laparoscopic ovarian cystectomy for endometriotic cysts in 132 monitored cycles Fertil Steril 1999 72 2 316 321 10.1016/S0015-0282(99)00207-1 10439003

[B9] Horikawa T Nakagawa K Ohgi S Kojima R Nakashima A Ito M The frequency of ovulation from the affected ovary decreases following laparoscopic cystectomy in infertile women with unilateral endometrioma during a natural cycle J Assist Reprod Genet 2008 25 6 239 244 10.1007/s10815-008-9229-y 18563551 PMC2582085

[B10] Shah DK Diminished ovarian reserve and endometriosis: insult upon injury Semin Reprod Med 2013 31 2 144 149 23446861 10.1055/s-0032-1333479

[B11] Tinkanen H Kujansuu E In vitro fertilization in patients with ovarian endometriomas Acta Obstet Gynecol Scand 2000 79 2 119 122 10.1034/j.1600-0412.2000.079002119.x 10696959

[B12] Donnez J Wyns C Nisolle M Does ovarian surgery for endometriomas impair the ovarian response to gonadotropin? Fertil Steril 2001 76 4 662 665 10.1016/S0015-0282(01)02011-8 11591395

[B13] Opøien HK Fedorcsak P Omland AK Abyholm T Bjercke S Ertzeid G Oldereid N Mellembakken JR Tanbo T In vitro fertilization is a successful treatment in endometriosis-associated infertility Fertil Steril 2012 97 4 912 918 10.1016/j.fertnstert.2012.01.112 22341637

[B14] Demirol A Guven S Baykal C Gurgan T Effect of endometrioma cystectomy on IVF outcome: a prospective randomized study Reprod Biomed Online 2006 12 5 639 643 10.1016/S1472-6483(10)61192-3 16790114

[B15] Steer CV Campbell S Pampiglione JS Kingsland CR Mason BA Collins WP Transvaginal color flow imaging of the uterine arteries during the ovarian and menstrual cycles Hum Reprod 1990 5 4 391 395 2193940 10.1093/oxfordjournals.humrep.a137109

[B16] Steer CV Tan SL Mason BA Campbell S Midluteal-phase vaginal color Doppler assessment of uterine artery impedance in a subfertile population Fertil Steril 1994 61 1 53 58 8293844 10.1016/s0015-0282(16)56452-8

[B17] Exacoustos C Brienza L Di Giovanni A Szabolcs B Romanini ME Zupi E Adenomyosis: three-dimensional sonographic findings of the junctional zone and correlation with histology Ultrasound Obstet Gynecol 2011 37 4 471 479 10.1002/uog.8900 21433167

[B18] El-Mazny A Abou-Salem N Elshenoufy H Doppler study of uterine hemodynamics in women with unexplained infertility Eur J Obstet Gynecol Reprod Biolin press 10.1016/j.ejogrb.2013.08.026 24011380

[B19] Guerriero S Ajossa S Mais V Risalvato A Lai MP Melis GB The diagnosis of endometriomas using color Doppler energy imaging Hum Reprod 1998 13 6 1691 1695 10.1093/humrep/13.6.1691 9688414

[B20] Joyce CR Zutshi DW Hrubes V Mason RM Comparison of fixed interval and visual analogue scales for rating chronic pain Eur J Clin Pharmacol 1975 8 6 415 420 10.1007/BF00562315 1233242

[B21] Manganaro L Fierro F Tomei A Irimia D Lodise P Sergi ME Feasibility of 3.0T pelvic MR imaging in the evaluation of endometriosis Eur J Radiol 2012 81 6 1381 1387 10.1016/j.ejrad.2011.03.049 21497034

[B22] Manganaro L Vittori G Vinci V Fierro F Tomei A Lodise P Sollazzo P Beyond laparoscopy: 3-T magnetic resonance imaging in the evaluation of posterior cul-de-sac obliteration Magn Reson Imaging 2012 30 10 1432 1438 10.1016/j.mri.2012.05.006 22835943

[B23] Manganaro L Vinci V Bernardo S Storelli P Fuggetta E Sollazzo P The role of 3.0T MRI in the assessment of deep endometriosis located on the uterosacral ligaments J Endom 2013 5 1 10 16

[B24] Kurjak A Kupesic-Urek S Schulman H Zalud I Transvaginal color flow Doppler in the assessment of ovarian and uterine blood flow in infertile women Fertil Steril 1991 56 5 870 873 1936320 10.1016/s0015-0282(16)54657-3

[B25] Kennedy S Bergqvist A Chapron C D’Hooghe T Dunselman G Greb R Hummelshoj L Prentice A Saridogan E ESHRE Special Interest Group for Endometriosis and Endometrium Guideline Development Group ESHRE guideline for the diagnosis and treatment of endometriosis Hum Reprod 2005 20 10 2698 2704 10.1093/humrep/dei135 15980014

[B26] Porpora MG Pallante D Ferro A Crisafi B Bellati F Benedetti Panici P Pain and ovarian endometrioma recurrence after laparoscopic treatment of endometriosis: a long-term prospective study Fertil Steril 2010 93 3 716 721 10.1016/j.fertnstert.2008.10.018 19061997

[B27] Osuga Y Koga K Tsutsumi O Yano T Maruyama M Kugu K Momoeda M Taketani Y Role of laparoscopy in the treatment of endometriosis-associated infertility Gynecol Obstet Invest 2002 53 Suppl 1 33 39 11834866 10.1159/000049422

[B28] Porpora MG Pultrone DC Bellavia M Franco C Crobu M Cosmi EV Reproductive outcome after laparoscopic treatment of endometriosis Clin Exp Obstet Gynecol 2002 29 4 271 273 12635743

[B29] Garcia-Velasco JA Somigliana E Management of endometriomas in women requiring IVF: to touch or not to touch Hum Reprod 2009 24 3 496 501 19056774 10.1093/humrep/den398

[B30] Benaglia L Somigliana E Vighi V Ragni G Vercellini P Fedele L Rate of severe ovarian damage following surgery for endometriomas Hum Reprod 2010 25 3 678 682 10.1093/humrep/dep464 20083485

[B31] Benaglia L Pasin R Somigliana E Vercellini P Ragni G Fedele L Unoperated ovarian endometriomas and responsiveness to hyperstimulation Hum Reprod 2011 26 6 1356 1361 10.1093/humrep/der097 21478182

[B32] Almog B Shehata F Sheizaf B Tan SL Tulandi T Effects of ovarian endometrioma on the number of oocytes retrieved for in vitro fertilization Fertil Steril 2011 95 2 525 527 10.1016/j.fertnstert.2010.03.011 20378109

[B33] Somigliana E Vercellini P Viganó P Ragni G Crosignani PG Should endometriomas be treated before IVF-ICSI cycles? Hum Reprod Update 2006 12 1 57 64 16155094 10.1093/humupd/dmi035

[B34] Benaglia L Somigliana E Iemmello R Colpi E Nicolosi AE Ragni G Endometrioma and oocyte retrieval-induced pelvic abscess: a clinical concern or an exceptional complication? Fertil Steril 2008 89 5 1263 1266 10.1016/j.fertnstert.2007.05.038 18339383

[B35] Benaglia L Somigliana E Vighi V Nicolosi AE Iemmello R Ragni G Is the dimension of ovarian endometriomas significantly modified by IVF-ICSI cycles? Reprod Biomed Online 2009 18 3 401 406 10.1016/S1472-6483(10)60099-5 19298740

[B36] Nezhat C Winer WK Cooper JD Nezhat F Nezhat C Endoscopic infertility surgery J Reprod Med 1989 34 2 127 134 2522547

[B37] Vercellini P Fedele L Aimi G De Giorgi O Consonni D Crosignani PG Reproductive performance, pain recurrence and disease relapse after conservative surgical treatment for endometriosis: the predictive value of the current classification system Hum Reprod 2006 21 10 2679 2685 10.1093/humrep/del230 16790608

[B38] Hart RJ Hickey M Maouris P Buckett W Garry R Excisional surgery versus ablative surgery for ovarian endometriomata Cochrane Database Syst Rev 2008 2 CD004992 10.1002/14651858.CD004992.pub2 16034960

[B39] Raine-Fenning NJ Campbell BK Kendall NR Clewes JS Johnson IR Endometrial and subendometrial perfusion are impaired in women with unexplained subfertility Hum Reprod 2004 19 2605 2614 10.1093/humrep/deh459 15465835

[B40] Maneschi F Marasá L Incandela S Mazzarese M Zupi E Ovarian cortex surrounding benign neoplasms: a histologic study Am J Obstet Gynecol 1993 169 2 Pt 1 388 393 8362952 10.1016/0002-9378(93)90093-x

[B41] Santoro L D’Onofrio F Campo S Ferraro PM Tondi P Campo V Flex A Gasbarrini A Santoliquido A Endothelial dysfunction but not increased carotid intima-media thickness in young European women with endometriosis Hum Reprod 2012 27 5 1320 1326 10.1093/humrep/des062 22416009

